# Assessment of Thermal Stability of Mutant p53 Proteins via Differential Scanning Fluorimetry

**DOI:** 10.3390/life13010031

**Published:** 2022-12-22

**Authors:** Raniya Khadiullina, Regina Mirgayazova, Damir Davletshin, Elvina Khusainova, Vitaly Chasov, Emil Bulatov

**Affiliations:** 1 Institute of Fundamental Medicine and Biology, Kazan Federal University, 420008 Kazan, Russia; 2 Shemyakin-Ovchinnikov Institute of Bioorganic Chemistry, Russian Academy of Sciences, 117997 Moscow, Russia

**Keywords:** p53, transcription factor, mutant, melting temperature, differential scanning fluorimetry

## Abstract

The p53 protein is a transcription factor that preserves the integrity of the genome. The *TP53* gene has inactivating mutations in about 50% of all human cancers. Some missense mutations lead to decreased thermal stability in the p53 protein, its unfolding and aggregation under physiological conditions. A general understanding of the impact of point mutations on the stability and conformation of mutant p53 is essential for the design and development of small molecules that target specific p53 mutations. In this work, we determined the thermostability properties of some of the most common mutant forms of the p53 protein—p53(R273H), p53(R248Q), p53(R248W) and p53(Y220C)—that are often considered as attractive therapeutic targets. The results showed that these missense mutations lead to destabilization of the p53 protein and a decrease in its melting temperature.

## 1. Introduction

**Figure 1:**
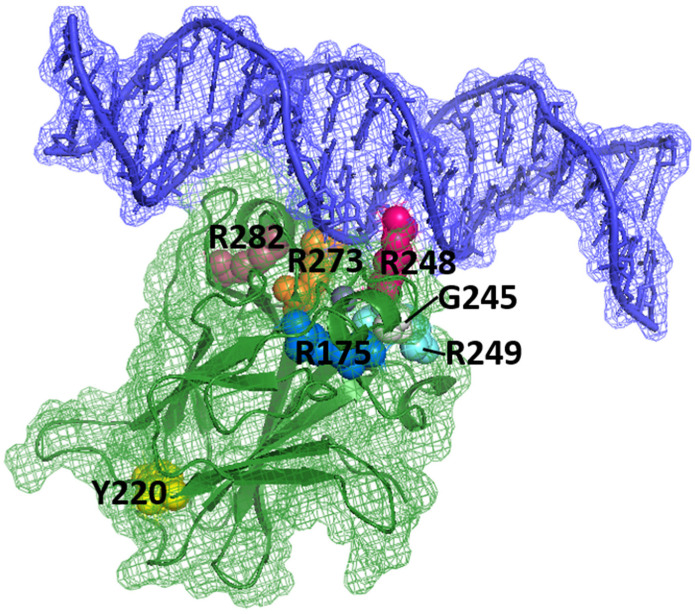
p53 DBD (green) in complex with DNA (blue). The locations of amino acids R175, Y220, G245, R248, R249, R273 and R282, which are sites of key carcinogenic mutations (“hot spots”), are shown (PDB identifier: 1TSR) [4]. DNA is in direct interaction with R273 and R248, and several “hot spots” are present near this interaction (R249, G245, R282). Other “hot spots” are far from the surface involved in DNA binding, for example, Y220.

The p53 tumor suppressor plays a key role in multiple signaling pathways that control cell cycle and is responsible for the stability of the human genome. Transcription factor p53 dysfunction occurs in most human malignancies [1]. Two main causes are responsible for this dysfunction: mutations in the *TP53* gene and suppression of wild-type p53 mediated by its negative regulators MDM2/MDM4. The p53 protein is inactivated by mutations in about half of all tumors [2]. Most p53 oncogenic mutations are missense mutations in the DNA-binding domain (DBD) of the protein [3]. Despite functional heterogeneity, these mutations can be broadly classified into contact and structural (Figure 1). The former disrupts the binding of p53 to DNA and shows relatively small changes in the overall structure and stability of the protein but contributes to the loss of its transcriptional activity (R248Q, R273H, R248W, R248L, R273C) [4]. The latter reduces the thermal stability of the p53 protein to varying degrees, causing it to rapidly unfold and aggregate under physiological conditions. The most-common structural mutations in p53 are G245S, R175H, R282W and Y220C [5]. Over 40% of missense mutations in the DNA-binding domain of p53 occur at arginine residues, while glycine and cysteine, the next most-common residues to change, mutate up to five-times less often. No p53 missense mutation is found at a frequency greater than 6%, and the 10 most-common and best-studied hotspot mutants together account for approximately 25% of all p53 mutations. Overall, these mutations lead to more than 2.5 million new cases of cancer per year worldwide [6].

Most mutant p53 proteins, in addition to losing their transcriptional activity (loss of function, LOF), can also acquire new oncogenic properties (gain of function, GOF). The p53 oncoprotein has a dominant-negative effect on the remaining wild-type p53 proteins. This effect is mediated by hetero-oligomerization of the mutant protein with the wild-type protein, which leads to the appearance of functionally inactive tetrameric complexes [7]. Further, the mutant protein leads to uncontrolled cell proliferation, inhibits apoptosis, confers resistance to specific anticancer compounds, and promotes invasion and metastasis.

According to the data provided in the IARC *TP53* database, missense mutations R248Q, R248W and R273H are among the five most-frequent p53 mutations that lead to more than 630,000 newly diagnosed cases of cancer worldwide every year [8]. The R248Q oncogenic mutation is the second-most-common mutation in p53. It is found in many types of cancer, such as colorectal cancer, lymphoid and myeloid leukemia, breast and ovarian cancer. Changing arginine to glutamine at position 248 disrupts binding of p53 to DNA [9]. R273H and R248W are the third- and fourth-most-common hotspot mutations, respectively. They are most often observed in lung, breast and ovarian cancer, as well as gliomas. Mutant proteins p53(R273H) and p53(R248W) not only lose their DNA-binding abilities but can also acquire new oncogenic properties that promote proliferation, chemoresistance and metastasis of tumor cells [10].

The oncogenic Y220C mutation is found in approximately 100,000 cancer cases each year [11]. This mutation changes the tertiary structure of the p53 DNA-binding domain, as a result of which the protein becomes destabilized, partially denatured and loses its cellular activity [8,12,13].

A decrease in the melting temperature (Tm) of the p53 DNA-binding domain is observed in 30% of all missense mutations that inactivate the protein [9,14]. Thus, the purpose of this work was to determine the melting temperature of the most-common mutant forms of p53.

## 2. Materials and Methods

### 2.1. Reagents

2-[4-(2-hydroxyethyl)piperazin-1-yl]ethanesulfonic acid (HEPES), phenylmethylsulfonyl fluoride (PMSF) and fluorescent dye SYPRO Orange were purchased from Sigma-Aldrich (St. Louis, MO, USA). Kanamycin, chloramphenicol and lysozyme were purchased from PanReac AppliChem (Boca Raton, FL, USA). Isopropyl-β-D-thiogalactoside (IPTG) and β-mercaptoethanol were from Anatrace (Maumee, OH, USA). 2-Amino-2-(hydroxymethyl)propane-1,3-diol (Tris) and dithiothreitol (DTT) were from Thermo Fisher Scientific (Waltham, MA, USA).

### 2.2. Site-Directed Mutagenesis

Plasmids encoding the p53 DBD (residues 94–312) (wild-type and Y220C mutant) were kindly provided by Prof. Matthias Baud from the University of Southampton (UK). Plasmids encoding the p53 core domains with R248Q, R248W and R273H hotspot mutations were prepared by site-directed mutagenesis at Evrogen Ltd. (Moscow, Russia). The primers are listed in Table 1.

The presence of a mutation in the gene was confirmed by sequencing. Plasmids were transformed into *Escherichia coli* by the heat-shock method. The pET24A(+) vector containing the p53 DBD gene (wt/mut) was isolated using the Plasmid Midiprep 2.0 Kit (cat. no. BC124).

### 2.3. Recombinant Production and Purification of p53

Proteins of p53 DBD (residues 94–312) were overexpressed in *Escherichia coli* BL21 (DE3) pLysS cells in TB medium at 18–20 °C for 16 h. For the primary purification of proteins, Ni Sepharose 6 FastFlow affinity sorbent (GE Healthcare, Chicago, IL, USA) was used. Proteolytic cleavage of hexa-histidine residues of recombinant proteins was performed using TEV protease. Further purification was carried out with HiTrap Heparin HP column (GE Healthcare, Chicago, IL, USA). Finally, size-exclusion chromatography was performed using ENrich SEC 70 column (Bio-Rad, Hercules, CA, USA) using an NGC Discover chromatography system (Bio-Rad, Hercules, CA, USA). The proteins were concentrated using a Vivaspin concentrator with molecular-weight cutoff of 10 kDa. Molecular weight and protein purity were confirmed by SDS gel electrophoresis.

### 2.4. Differential Scanning Fluorimetry (DSF)

The method is based on measuring the optical density (λ = 570 nm) of a gradually heated solution containing the protein and the hydrophobic fluorescent dye Sypro Orange (Sigma-Aldrich, St. Louis, MO, USA). The unfolding of the protein with increasing temperature and the opening of its hydrophobic regions leads to release of fluorescent signal that is recorded in a characteristic graph [14,15]. The experiment was carried out in 96-well LightCycler 480 Multiwell Plate 96 plates (Roche Diagnostics GmbH, Penzberg, Germany). Protein (final concentration of 10 µM) was mixed with SyproOrange fluorescent dye (up to ×10) (λex = 470 nm, λem = 570 nm) in 25 mM Hepes buffer pH = 7.2, 150 mM NaCl. The fluorescence readings were carried out using Lightcycler 480 thermal cycler (Roche, Penzberg, Germany), in which the plate was smoothly heated from 30 °C to 95 °C at a constant heating rate of 1 °C/min. The excitation and emission filters were set at λ = 460 nm and λ = 510 nm, respectively. All measurements were carried out in triplicate.

## 3. Results and Discussion

To determine the impact of missense mutations on p53 thermostability, the melting temperatures of the recombinant core domains p53(wt), p53(Y220C), p53(R248Q), p53(R248W) and p53(R273H) were studied via DSF. This technique allows for rapid analysis of protein stability in solutions [16,17]. Thermostable fluorescent dyes can bind nonspecifically to the hydrophobic surface of the protein. At a gradual increase in temperature, the protein thermal denaturation causes unfolding of the hydrophobic core, making it accessible to the dye molecules. When the dye binds to the hydrophobic core, this eliminates fluorescence quenching, and the fluorescence quantum yield increases dramatically. The optical signal is measured at each point and the thermal denaturation curve is generated, which allows for calculation of the melting temperature of a protein globule and, therefore, estimation of the protein thermal stability [18,19].

**Figure 2:**
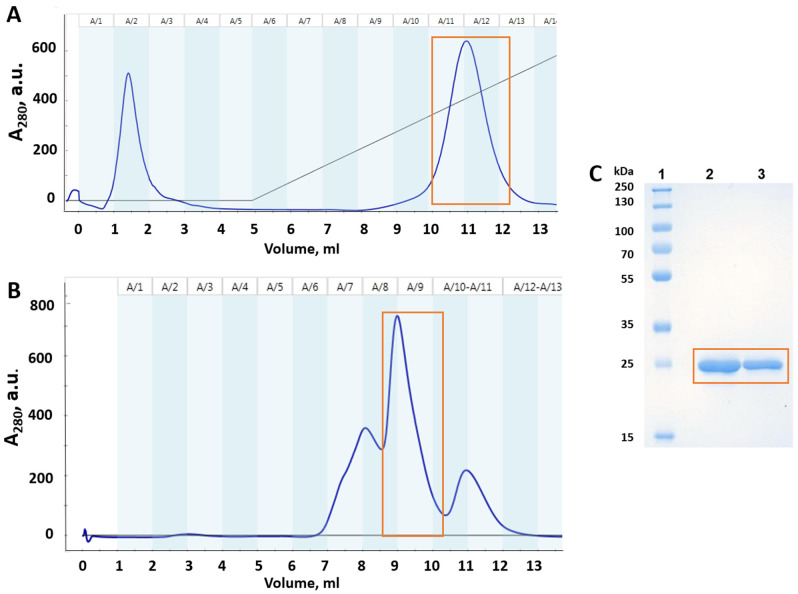
Purification of mutant p53(Y220C) DBD. Orange square shows the purified mutant p53(Y220C) DBD protein: (**A**) heparin-sepharose chromatography profile (dark blue line—absorbance at 280 nm); (**B**) gel filtration chromatography profile; (**C**) SDS-PAGE monitoring of mutant p53(Y220C) DBD after gel filtration chromatography: Lane 1—Protein molecular-weight marker; Lane 2—p53(Y220C) DBD (10 µg/well); Lane 3—p53(Y220C) DBD (5 µg/well).

The full-length p53 mutants are known to be thermodynamically unstable, prone to aggregation and have a lot of disordered N- and C-terminal parts [19]. The p53 core domain is key to protein stability, and mutations in this domain directly affect relative stability of the whole protein [20]. Therefore, in this study, we expressed and purified recombinant p53 DBD for wild-type and mutated isoforms. Representative chromatograms and electropherograms of purified p53(Y220C) DBD are shown in Figure 2. The degree of electrophoretic homogeneity of recombinant protein was above 95%, according to the results of densitometric analysis (Image Lab 6.0.1, Bio-Rad, Hercules, CA, USA).

**Figure 3:**
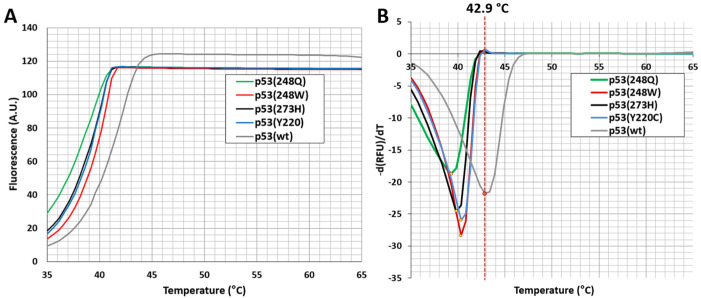
Analysis of the thermal stability of DNA-binding domains (residues 94–312) of p53 (wild-type) and p53(Y220C), p53(R248Q), p53(R248W) and p53(R273H) mutants by differential scanning fluorimetry. (**A**) Representative melting curves and (**B**) derivative curves. Protein (at final concentration of 10 µM) was mixed with SyproOrange fluorescent dye (up to ×10) in 25 mM Hepes buffer pH = 7.2, 150 mM NaCl. Colored curves represent the thermal denaturation patterns of various p53 mutants—p53(Y220C) in light blue, p53(R248Q) in green, p53(R248W) in red and p53(R273H) in black. The red vertical dashed line indicates the Tm of p53 (wild-type) DBD (gray curve). RFU is a relative unit of fluorescence.

According to the melting curves of the wild-type p53 and its four mutant variants, the wild-type protein exhibits the highest thermal stability among them (Figure 3). The Tm of the wild-type p53 DBD was around 42.9 °C, which is in agreement with the previously reported data (Table 2) [16,20,21,22,23]. The Tm values of DNA contact mutants p53(R248Q), p53(R248W) and p53(R273H) were equal to 38.5 °C, 39.3 °C and 38.8 °C, respectively (Figure 3 and Table 2).

Structural mutation of p53(Y220C), which is the ninth-most-common p53 mutation, reduces the stability of p53 DBD by 4 kcal/mol [24]. This results in unfolding and transcriptional inactivation of over 80% of the protein under physiological conditions. According to our data, the Tm value of p53(Y220C) mutant was equal to 40.3 °C (Figure 3 and Table 2).

We demonstrate that p53 mutations Y220C, R248Q, R248W and R273W destabilize the protein and the results are consistent with earlier studies [12,25,26,27,28,29]. Most DNA contact mutations are structurally conserved and normally do not have a profound effect on the p53 thermostability [30,31]. However, our data show that the difference in thermostability between DNA contact (R248Q, R248W and R273H) and structural (Y220C) mutations might be less pronounced than previously thought (ΔTm = approx. 1 °C). In support of this, some p53 mutations were indeed described to exhibit characteristics of both contact and structural mutations [10]. Tumor cells can contain a wide range of p53 mutants with reduced thermostability. The development of drugs capable of restoring wild-type function in such a heterogeneous target is, no doubt, a challenging task [32]. Alan Fersht and colleagues proposed several criteria that should be met by compounds considered as mutant p53 reactivators. Compounds should demonstrate binding to mutant p53 and lead to its thermal stabilization, restore the correctly folded state and activate the expression of p53 target genes in cancer cells [33]. Thus, the data on melting temperatures of various mutant forms of p53 allow for estimations of reactivation levels when screening small-molecule stabilizers.

## 4. Conclusions

Hotspot mutants of the p53 core domain are of considerable interest for the field of cancer research. In this study, we aimed to examine the effect of a series of missense p53 mutations on protein thermostability. For that, we determined the melting temperatures of wild-type, Y220C, R248Q, R248W and R273H variants of the p53 core domain via differential scanning fluorimetry. We confirmed that these missense mutations result in destabilization of the p53 protein and lower its melting temperature. Knowledge of the melting behavior of mutant p53 proteins is important for revealing the mechanisms of p53 inactivation and will further facilitate the development of personalized small-molecule therapeutics targeting p53 mutants.

## Figures and Tables

**Table 1:** Primer sequences for site-directed mutagenesis.

Mutant	Primer Sequences	Amino Acid Change: wt → Mutant
R248W	Rev: GTTCATGCCGCCCATGCAGGAACTGTAACACATGTAGTTGTAGTGGATGGT For: CCTGCATGGGCGGCATGAACtGGAGGCCCATCCTCACCATCATC	CGG (R) → TGG (W)
R248Q	Rev: GTTCATGCCGCCCATGCAGGAACTGTAACACATGTAGTTGTAGTGGATGGT For: CTGCATGGGCGGCATGAACCaGAGGCCCATCCTCACCATCATC	CGG (R) → CAG (Q)
R273H	Rev: GCACCTCAAAGCTGTCCCGTCCCAGTAGATTACCACTGGAGTCTTCC For: ACGGGACAGCTTTGAGGTGCaTGTTTGTGCCTGTCCTGGGAGA	CGT (R) → CAT (H)

**Table 2:** Melting temperatures of wild-type p53 and its mutant variants measured by differential scanning fluorimetry.

Protein	Structural Region	Tm, °C	ΔTm, °C *
p53 (wild-type)	-	42.9 ± 0.0	-
p53(Y220C)	β-sandwich	40.3 ± 0.2	−2.6
p53(R248Q)	DNA contact	38.5 ± 0.3	−4.4
p53(R248W)	DNA contact	39.3 ± 0.0	−3.6
p53(R273H)	DNA contact	38.8 ± 0.3	−4.1

* ΔTm = Tm (mutant) − Tm (wild-type). Mean values of triplicate measurements ± SEM are shown.

## Data Availability

Data related to this study can be provided by the corresponding authors on request.

## Abbreviations

DBD	DNA-binding domain
DSF	Differential scanning fluorimetry
MDM2	Mouse double minute 2 homolog
MDM4	Mouse double minute 4 homolog
NGC	Next-generation chromatography
PCR	Polymerase chain reaction
SDS	Sodium dodecyl sulfate
TB	Terrific broth
Tm	Melting temperature
WT	Wild type
